# Saline Permafrost and Cryopegs as Potentially Important Sources of CO_2_ —Assessing Organic Carbon Mineralization Potentials on the Alaskan Coastal Plain

**DOI:** 10.1111/gcb.70997

**Published:** 2026-07-11

**Authors:** Fabian Seemann, Mackenzie R. Baysinger, Susanne Liebner, Claire Treat, Michael Zech, Maren Jenrich, Guido Grosse, Benjamin M. Jones, Jens Strauss

**Affiliations:** ^1^ Permafrost Research Section Alfred Wegener Institute Helmholtz Centre for Polar and Marine Research Potsdam Germany; ^2^ Institute of Geosciences University of Potsdam Potsdam Germany; ^3^ Physical Geography, Institute of Geography Technische Universität Dresden Dresden Germany; ^4^ GFZ Helmholtz Centre for Geosciences, Section Geomicrobiology Potsdam Germany; ^5^ Institute of Biochemistry and Biology University of Potsdam Potsdam Germany; ^6^ Land‐CRAFT Center, Department of Agroecology Aarhus University Aarhus Denmark; ^7^ Institute of Northern Engineering University of Alaska Fairbanks Fairbanks Alaska USA

**Keywords:** aerobic incubation, Arctic Alaska, carbon pools, continuous permafrost zone, cryopeg, *n*‐alkane biomarkers, refrozen permafrost, saline permafrost, thermokarst

## Abstract

Thermokarst lake and drained lake basin (DLB) dynamics are intensifying across the Alaskan Arctic Coastal Plain. Thawing, drainage, and erosion expose surface and deep sediments (> 1 m) to aerobic conditions, with saline deposits being particularly vulnerable due to freeze‐point depression. As organic carbon mineralization remains poorly constrained, we determined potentials with an aerobic one‐year long incubation at 10°C in permafrost upland, lake talik, lake cryopeg, and refrozen saline DLB sediments. We linked CO_2_ production to biochemical, hydrochemical, and microbial factors, and assessed carbon alteration via repeated *n*‐alkane analyses. After 382 days, average CO_2_ production was 7.0 ± 0.4 mg C g^−1^ dry weight (DW), with DLB surface peat yielding the most (40.5 ± 2.7 mg C g^−1^ DW), and carbon‐poor cryopeg deposits (1.4 ± 0.1 mg C g^−1^ DW) and refrozen saline permafrost (1.5 ± 0.02 mg C g^−1^ DW) the least. Total organic carbon (TOC) was the main driver of CO_2_ production, while age, nitrogen content, electrical conductivity, water content, pH, and microbial abundance also correlated significantly with CO_2_ production. Normalizing production to TOC contents, saline permafrost and cryopeg sediments showed similar CO_2_ production to active layers, stressing the importance of potentially carbon‐rich saline deposits. TOC normalization revealed that carbon characteristics (δ^13^C, alkane content, ACL) also significantly influenced CO_2_ production. The *n*‐alkane based quantification of carbon alterations during the incubation further contributes to the understanding of carbon cycling at the molecular level. *n*‐Alkane contents increased on average by 153% and the carbon preference index (CPI) rose from 13.2 to 15.8, likely due to newly produced alkanes, preferential degradation, and desorption processes. This indicates strong responses of the carbon pool and raises questions about the reliability of the CPI as a degradation proxy. Altogether, our study highlights the overlooked role of salinity in CO_2_ production from Arctic coastal plains which could substantially shift carbon balances.

## Graphical abstract

Incubation experiments reveal that potentially carbon‐rich saline permafrost and cryopegs from Arctic coastal plains exhibit CO_2_ production potentials comparable to active layers. This could substantially shift greenhouse gas balances, as salinity is currently not accounted for in permafrost carbon assessments. CO_2_ production was primarily driven by organic carbon quantity, while carbon quality further modulated decomposition rates. In addition to bulk carbon analyses, *n*‐alkane biomarkers proved effective for tracing carbon cycling at the molecular scale.

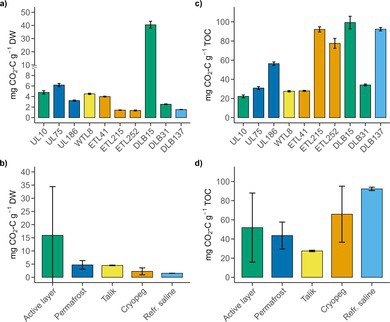

## 1 Introduction

Arctic coastal lowlands are undergoing rapid changes as Arctic warming exceeds global average rates and intensifies coastal erosion processes (Irrgang et al. 2022; Nielsen et al. 2022; Rantanen et al. 2022). Warming in such ice‐rich landscapes leads to gradual active layer deepening (Fox‐Kemper et al. 2021; Liu et al. 2024; Streletskiy et al. 2025), and abrupt thaw processes such as thermokarst, a process driven by melt of excess ice and associated thaw subsidence (Kokelj and Jorgenson 2013; Webb et al. 2025). On short timescales abrupt thaw reaches deeper sediments compared to gradual thaw (Webb et al. 2025), creating lowlands with scattered thermokarst lakes (Jones et al. 2022). Unfrozen sediments beneath thermokarst lakes are referred to as subaquatic taliks, which typically refreeze under exposed basins when lakes drain. However, with continued warming, taliks are projected to incompletely refreeze in the continuous permafrost zone (Farquharson et al. 2022; Jones et al. 2022; Lantz et al. 2022). Moreover, large parts of Arctic coastal lowlands are underlain by saline deposits of marine origin, where salt concentrations result in depressing the freezing point of water, thereby enabling permafrost thaw well below 0°C (Brouchkov 2003; Hinkel et al. 2005; Lyu et al. 2025). If such saline sediments occur under unfrozen cryotic conditions, they are defined as cryopegs (Irrgang et al. 2022). With ongoing climate warming, the presence and extent of cryopegs can be expected to increase (Jones et al. 2023; Seemann et al. 2026).

The cumulative impact of these factors and processes is critical to Arctic permafrost landscapes and their role in the global climate system, since thermokarst landscapes store a major proportion of the total soil organic carbon pool (Olefeldt et al. 2016; Strauss et al. 2025). Recent research shows that permafrost carbon emissions and climate change relate almost linearly on the global scale (Nitzbon et al. 2024). However, on regional to local scales, abrupt non‐linear processes are important, and especially such dynamics found along the Arctic Ocean, where coastlines are eroding rapidly and saline deposits are present, are currently not sufficiently resolved in global‐scale models (Creel et al. 2024; Schädel et al. 2024; Turetsky et al. 2020).

As permafrost thaws, microbial decomposition converts organic carbon into carbon dioxide (CO_2_) and methane (CH_4_). The relative proportion of these greenhouse gases (GHGs) produced during degradation is governed by microbial community composition and redox conditions. While CO_2_ is the dominant product of respiration under aerobic conditions, both CO_2_ and CH_4_ are produced through fermentation and methanogenesis in anaerobic environments (Knoblauch et al. 2013; Schuur et al. 2015; Treat et al. 2014).

Carbon emissions of thermokarst landscapes may be assessed by various methodical approaches. Eddy covariance systems are used to monitor GHG fluxes on the landscape level (Dengel et al. 2021), whereas chamber systems are frequently used to quantify GHG fluxes from specific landforms (Laurion et al. 2010). In laboratory‐based experiments, sediment samples can be incubated under controlled conditions, allowing specific sediment types and thermal, physical and redox regimes to be isolated, thereby improving process understanding and quantifying GHG potentials. Except for the surface sediments, anaerobic conditions often prevail in thermokarst landforms (Dolle et al. 2025; in't Zandt et al. 2020). Yet, lake drainage and coastal erosion may lead to the exposure of deep sediments to well aerated conditions (Tanski et al. 2019). It is therefore essential to experimentally assess organic carbon mineralization potentials of coastal lowlands under aerobic conditions.

Previous aerobic incubation experiments have focused on a large range of factors, particularly emphasizing organic carbon characteristics (Lee et al. 2012), but also including factors like sediment age/origin (Knoblauch et al. 2021), landform (Herbst et al. 2024; Tanski et al. 2019), and thermal regime (Chen et al. 2016; Walz et al. 2018). The role of salinity has been addressed by artificially adding seawater to sediment in anaerobic conditions, simulating inundation, coastal erosion and lagoon formation (Dolle et al. 2025; Guimond et al. 2021; Jenrich et al. 2024, 2025; Ruben et al. 2024). To the best of our knowledge, Tanski et al. (2019, 2021) are the only studies to have investigated the role of salinity in aerobic environments with the addition of seawater, and they found greater CO_2_ production than in non‐saline samples. Since specific studies on sediments that are in situ saline (including cryopegs) are missing, their response to thawing under aerobic conditions remains unknown. Thus, the overarching aim of our study is to fill this gap by conducting an aerobic incubation experiment with representative sediments from thermokarst terrain of the Alaskan Coastal Plain.

The lability of organic carbon has been determined as a key factor for microbial carbon mineralization in permafrost thaw settings (Dutta et al. 2006). Next to bulk organic matter quality proxies such as C:N (Schädel et al. 2014), *n*‐alkane biomarkers provide detailed insights into organic carbon characteristics on the molecular level (Jongejans et al. 2020). Through degradation processes, it has been shown that total alkane contents decrease (Thomas et al. 2021). Moreover, a prominent *n*‐alkane proxy is the carbon preference index, describing the ratio between odd and even alkane chain lengths, which decreases with advancing organic matter decay (Marzi et al. 1993). *n*‐Alkanes have been analyzed in the context of GHG production prior to incubation in order to characterize organic matter (Jongejans et al. 2021). However, since the possibility of repeating *n*‐alkane analyses after incubations in permafrost contexts has to date not yet been applied, it is unknown how *n*‐alkane patterns change after thaw and microbial use, although such investigations are crucial to better resolve the fate of organic carbon. Therefore, we further aimed at investigating the driving factors of carbon mineralization by established sedimentological, hydrochemical, and microbial analyses before reinvestigating *n*‐alkane proxies.

Based on the aforementioned described research gaps, we defined the following three research questions for our study:

1. How do CO_2_ production potentials vary across thermokarst terrain on the Alaskan Coastal Plain, differing in sediment properties and thermal regimes, including active layers, undisturbed non‐saline permafrost, a lake talik, a lake cryopeg, and refrozen saline permafrost in a drained lake basin?
2. What are the driving factors for observed CO_2_ productions?
3. How do *n*‐alkane patterns alter during organic carbon mineralization?

## 2 Material and Methods

### 2.1 Study Area

**FIGURE 1:**
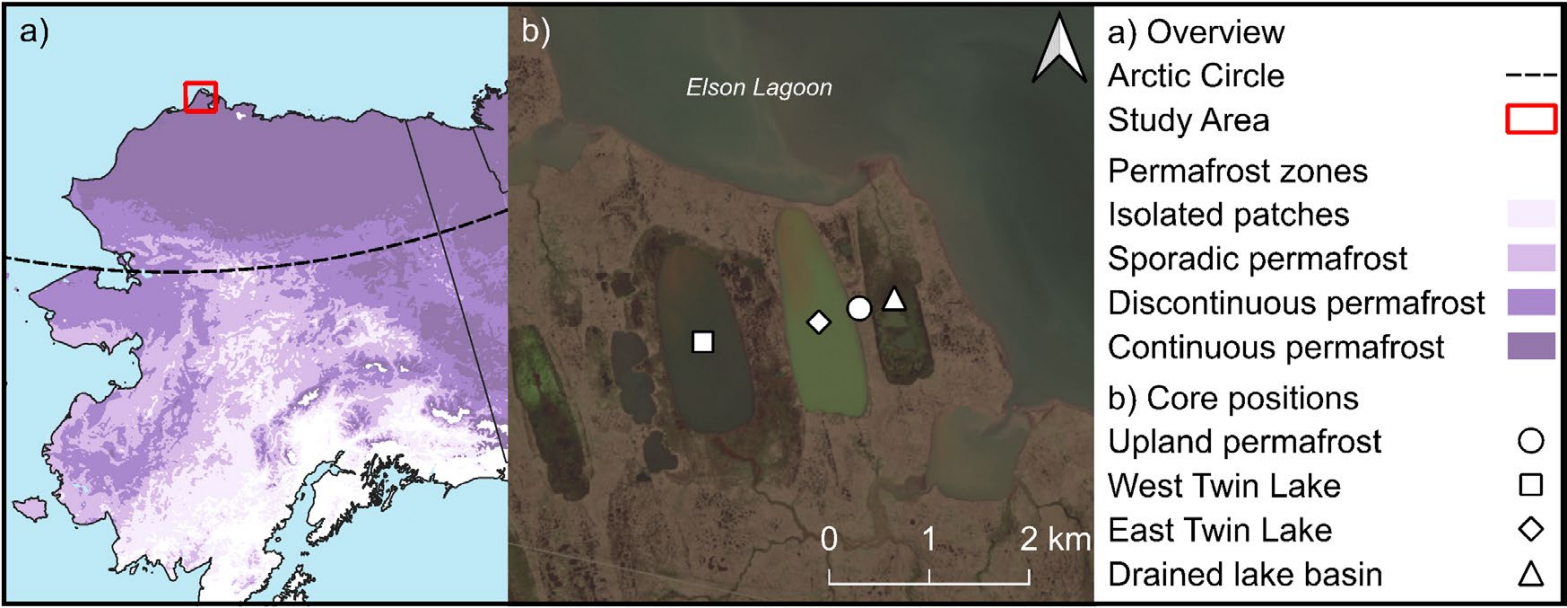
(a) Location of the study area in the continuous permafrost zone of Alaska (map based on data from Obu et al. 2018). (b) Coring positions in the study area (Sentinel‐2 satellite image from Copernicus 2023). Map lines delineate study areas and do not necessarily depict accepted national boundaries.

The study area is located ~10 km east of Utqiaġvik in northernmost Alaska (71.276° N, 156.453° W; ca. 2–5 m a.s.l.; Figure 1).

Located in the continuous permafrost zone, mean annual ground temperatures at the top of permafrost are around −6°C and the permafrost thickness reaches at maximum ~400 m (Brown et al. 2003; Jorgenson et al. 2008; Obu et al. 2019). The active layer depth ranged between 29 and 47 cm during the period 1995–2019 (Nyland et al. 2021). Between 2016 and 2020, the mean annual air temperature was −7.8°C and annual precipitation averaged 200 mm, both exhibiting significant increasing trends since 1981 (Rawlins 2021).

The study region lies within the Younger Outer Coastal Plain of the Arctic Coastal Plain (Hinkel et al. 2003). Saline deposits within the landscape reflect past marine inundations during higher sea level stands which occurred repeatedly during the late Cenozoic. In particular, most recent transgressions during Marine Isotope Stage 5a/4 (ca. 58–75 ka) where local sea levels were about 7 m higher than today, and MIS 5e (ca. 123 ka) with sea levels 10–13 m higher than today have contributed to the stratigraphy, reflecting complex relative sea level changes in the region. These marine‐derived sediments were subsequently preserved under permafrost conditions, leading to the occurrence of saline permafrost (Brigham‐Grette and Hopkins 1995; Dinter et al. 1990; Eisner et al. 2005; Hinkel et al. 2005).

The landscape is characterized by ice wedge polygonal tundra (Eisner et al. 2005). Surface sediments consist of ice‐ and organic‐rich Holocene deposits with a thickness of less than 1.5 m overlaying the saline sediments (Seemann et al. 2026). Thermokarst features dominate the landscape, with ~50% of the area comprising drained lake basins (DLBs) and ~22% covered by thermokarst lakes (Hinkel et al. 2003; Jones et al. 2022). For our study lakes, Jones et al. (2023) observed freshwater conditions in the 1.81 m deep West Twin Lake, while East Twin Lake is a 1.75 m shallow brackish thermokarst lake actively thawing sub‐lake saline permafrost. As the latter retains unfrozen lake sediments at temperatures below 0°C, these sediments are defined as a cryopeg (Jones et al. 2023; Seemann et al. 2026). The DLB investigated in this study likely drained ~700 cal a BP due to coastal erosion (Seemann et al. 2026), initiating permafrost re‐aggradation and ice wedge polygon formation (Andresen and Lougheed 2015). Erosion rates along Elson Lagoon in the north and east of the study area range between 0.3–5 m yr^−1^. (Gibbs and Richmond 2017; Zimmermann et al. 2022).

### 2.2 Methods

#### 2.2.1 Sediment Sampling

Four sediment cores were collected during fieldwork in April 2022. The sampling design followed a transect covering a space‐for‐time permafrost degradation gradient (undegraded upland permafrost, freshwater and brackish lake deposits, refrozen drained lake basin deposits). The upland (204 cm recovery depth) as well as the DLB (142 cm recovery depth) core were collected with a 7.62 cm (3″) diameter snow, ice and permafrost (SIPRE) auger. West Twin Lake was sampled with a 7 cm (2.75″) diameter pushcorer (23 cm recovery depth) and East Twin Lake (259 cm recovery after correcting for compression following Seemann et al. 2026) with a 7.5 cm (2.95″) diameter vibration coring system.

After frozen (upland and DLB core) and unfrozen (lake cores) sample transport to AWI (Potsdam, Germany), the frozen and unfrozen cores were stored and processed at −8°C and 4°C, respectively. Cores were split in half, with one half being archived. Subsamples along the cores were divided in adjusted steps following the sediment stratigraphy and structure. Sample names were standardized using abbreviations of the landforms (UL—upland, WTL—West Twin Lake, ETL—East Twin Lake, DLB—drained lake basin) and the mean sample depth below sediment surface in cm. Subsequently, the individual samples were weighed and processed for sedimentological and biogeochemical analysis.

#### 2.2.2 Sediment Characteristics

Porewater was extracted from sister samples using Rhizon samplers (0.12–0.18 μm membrane pore size). Electrical conductivity (EC; mS cm^−1^) and pH were determined with an Orion VERSASTAR PRO (Thermo Fisher). Sediment samples were subsequently freeze‐dried. The calculated absolute soil water content (SWC) is expressed in weight percent (wt%).

Subsamples were milled using a planetary mill (Fritsch PULVERISETTE 5). Total organic carbon (TOC) and total nitrogen (TN) contents were measured using a soliTOC and rapid N exceed analyzer (Elementar Analysensysteme). Although instrument detection limits are 0.001 wt% (TOC) and 0.05 wt% (TN), we applied a conservative threshold of 0.1 wt% for both to avoid misinterpreting low values as absence of material, following Strauss et al. (2022). C:N ratios were only calculated when TN exceeded the detection limit to avoid artifacts.

Organic matter ages were determined using radiocarbon (^14^C) dating, which was performed at the Alfred Wegener Institute's MICADAS facility (Mollenhauer et al. 2021). Radiocarbon ages were calibrated using Calib 8.20 and the IntCal20 calibration curve (Reimer et al. 2020; Stuiver and Reimer 1993).

Stable carbon isotope ratios (δ^13^C) are widely used to trace organic matter origin and degradation in permafrost regions (Strauss et al. 2015). Samples were measured on a Thermo Fisher Delta‐V‐Advantage IRMS with FLASH EA 2000 and CONFLO IV. Results are reported in ‰ relative to the Vienna Pee Dee Belemnite (Coplen et al. 2006). All sedimentological and hydrochemical data is published by Seemann, Baysinger, Liebner, et al. (2025), Seemann, Jenrich, Lindemann, et al. (2025a), Seemann, Jenrich, Lindemann, et al. (2025b), and Seemann, Liebner, Saborowski, Jenrich, et al. (2025).

#### 2.2.3 Incubation Experiment

An ex‐situ experimental incubation was conducted at 10°C under dark and aerobic conditions for 382 days. We chose 10°C as it approximately represents the maximum average daily air temperature (10.8°C in July 2025; Alaska Climate Research Center 2026). Such temperatures are likely to occur more regularly with ongoing climate warming in the study area. Using a higher temperature of the temperature range simulates conditions under which microbial activity is strongest (Rijkers et al. 2023). Aerobic conditions were applied, as lake drainage and erosion may lead to oxic conditions in the represented landscape positions. For a detailed investigation of GHG production we incubated samples for more than one year, which allows experimentally assessing long‐term responses rather than short periods (Schädel et al. 2014). This approach amplifies organic carbon mineralization and GHG production processes, resolving biogeochemical transformations more clearly.

In total, ten samples were selected from various positions along the cores, representing on the one hand different thermal domains. On the other hand, multiple depths were chosen due to the underrepresentation of subsoils in incubation studies (Zhang et al. 2026). The sample set includes: the active layer (UL, DLB), upland permafrost (UL), talik (WTL), cryopeg (ETL), and refrozen permafrost (DLB). From every sample, three aliquots with each 15 g wet weight were homogenized and put into sterile vials (105 mL) closed with septa (in total 30 subsamples). Additionally, a blank was prepared for experimental control. At day 0 of the experiment, the vials were flushed with synthetic air (20% O_2_, 80% N_2_). CO_2_ and CH_4_ concentrations were measured three times per week (first two weeks), two times per week (week 3–7), one time per week (week 8–15), and in a regular two‐week interval until day 382 (week 55).

The septa of the vials were sterilized with inflamed 99% ethanol. Using a Hamilton gastight syringe, 250 μL headspace gas was injected into a gas chromatograph (7890A, Agilent Technologies, USA). CO_2_ and CH_4_ were separated over an Agilent 19095PQO4 column (helium as carrier gas) and measured by a flame ionization detector. If CO_2_ concentrations exceeded 10,000 ppm, the vial's headspace was immediately flushed with synthetic air for three minutes and re‐measured afterwards to maintain oxic conditions. After the experiment, the sediment of two replicates was used to conduct repeated biomarker analyses and the unopened third replica remains stored frozen in the archive.

Incubation raw data was treated in RStudio following the script of Baysinger and Dolle (2025). GHG production rates result from the difference of the gas concentration between measurement days (Robertson 1999). Blank measurements were treated as the minimum CO_2_ change rates. The raw ppm data was converted to mg CO_2_‐C by applying the Ideal Gas Law and considering the vial's headspace corrected with the sample volumes. As wet samples were incubated, Henry's Law was applied as well to account for dissolved gas. Production rates were finally normalized by per gram dry weight (DW) and TOC. The cumulative production was calculated as the sum of CO_2_ production during the incubation period. Methane production was only observed during the initial phase of the incubation (first ~8 days) and declined to near‐zero levels rapidly after. Given the continuously aerobic conditions, this transient CH_4_ signal most likely reflects outgassing of methane previously stored in pore spaces rather than sustained methanogenesis, and is therefore not further considered. The incubation data is accessible via Seemann, Baysinger, Liebner, et al. (2025), Seemann, Jenrich, Lindemann, et al. (2025a), Seemann, Jenrich, Lindemann, et al. (2025b), and Seemann, Liebner, Saborowski, Jenrich, et al. (2025).

#### 2.2.4 *n*‐Alkane Biomarker Analyses

*n*‐Alkane analyses were carried out before and after the incubation experiment using sister samples. The laboratory work was carried out following established procedures (Giest et al. 2025; Windirsch et al. 2025). In short, the total lipid extract of the samples was eluted with a Dionex ASE 350 Accelerated Solvent Extractor using dichloromethane/methanol. After adding the internal standard 5α‐androstane to the extract, asphaltenes were filtered. The aliphatic fraction was extracted by medium pressure liquid chromatography (Radke et al. 1980). Elemental sulfur was removed from the samples through a reaction with copper flakes. Subsequently, aliphatics were measured with a Thermo Scientific ISQ 7000 Single Quadrupole Mass Spectrometer coupled with a Thermo Scientific Trace 1310 Gas Chromatograph. Manual *n*‐alkane (nC_23_‐nC_33_) quantification was carried out with the Xcalibur software and results are published by Seemann, Baysinger, Liebner, et al. (2025), Seemann, Jenrich, Lindemann, et al. (2025a), Seemann, Jenrich, Lindemann, et al. (2025b), and Seemann, Liebner, Saborowski, Jenrich, et al. (2025).

During data evaluation, the total *n*‐alkane content (TAC; Equation 1), the average chain length (ACL; Equation 2), and the carbon preference index (CPI; Equation 3) were calculated. While TAC and CPI are considered indicative for organic carbon degradation, ACL reflects organic matter sources (Schäfer et al. 2016; Thomas et al. 2021). The CPI values of highly degraded substances approach 1, while values > 5 are considered as little degraded (Haugk et al. 2022; Killops and Killops 2005).

(1) TAC=ΣC23−C33

(2) $$\mathrm{ACL}\;23-33=\frac{\Sigma \;i\times \mathrm{Ci}}{\Sigma \;\mathrm{Ci}}$$

(3) $$\mathrm{CPI}\;23-33=\frac{\Sigma \;\mathrm{odd}\;C23-31+\Sigma \;\mathrm{odd}\;C25-33}{2\times \Sigma \;\text{even}\;C24-32}$$

#### 2.2.5 Microbiological Investigations: DNA Extraction and Quantification of Bacterial Abundance

During preparation of incubation vials, 1.5 g wet sample was filled into Eppendorf vials. Total nucleic acids were extracted in duplicate using the DNeasy PowerSoil Pro Kit (Qiagen, Germany) according to the manufacturer's instructions. DNA extracts were subsequently purified with the DNA Clean & Concentrator‐25 Kit (Zymo Research, USA). Bacterial abundance was estimated via quantitative PCR (qPCR) targeting the 16S rRNA gene, employing primers Eub341‐F (5′‐CCTACGGGAGGCAGCAG‐3′) and Eub534‐R (5′‐ATTACCGCGGCTGCTGG‐3′). Each qPCR reaction (20 μL) consisted of 2× SensiFAST SYBR Mix (Kapa Biosystems, Wilmington, USA) and 100 μM of each primer (Microsynth AG, Germany). Standard curves were generated from serial 1:10 dilutions of templates with known gene copy numbers, producing five standards that were analyzed in triplicate alongside the samples. A negative control containing PCR‐grade water was included in each run. The qPCR amplification was conducted on a Bio‐Rad CFX Connect system with the following cycling conditions: initial denaturation at 95°C for 3 min, 35 cycles of 95°C for 3 s, annealing at 60°C for 20 s, and extension at 72°C for 30 s, followed by a plate reading at 80°C for 3 s to prevent overestimation caused by primer dimers. A melting curve analysis was performed at the end of each run to verify product specificity and overall assay quality. Processes data is published in the PANGAEA data repository (Seemann, Liebner, Saborowski, Jenrich, et al. 2025).

#### 2.2.6 Statistical Analyses

All statistical analyses were conducted in R (version 4.1.2; R Core Team 2023) and significance was accepted at *p* < 0.05. Differences in CO_2_ production rates (per DW and TOC) among sediment samples and their positions were assessed using one‐way analysis of variance (ANOVA; Gotelli and Ellison 2013). Data were first inspected for normality and homogeneity of variances using the Shapiro–Wilk test (Shapiro and Wilk 1965) and Levene's test (Lewis‐Beck et al. 2004), respectively. When assumptions were met, ANOVA was followed by Tukey's honest significant difference (HSD) post hoc tests (Tukey 1949) to identify pairwise differences. In cases where assumptions were violated, data were log‐transformed and re‐tested; if normality could not be achieved, non‐parametric Kruskal–Wallis tests (Kruskal and Wallis 1952) were applied.

Furthermore, we tested for relationships between potential CO_2_ production and soil properties (TOC, TN, δ^13^C, SWC, pH, EC, TAC, ACL, CPI, 16S rRNA). As most variables, including cumulative CO_2_ production, deviated from normality (Shapiro–Wilk test, *p* ≤ 0.05), Spearman's rank correlation coefficient (*ρ*) was used to assess monotonic relationships between variables (Spearman 1904). Moreover, a principal component analysis (PCA; Gotelli and Ellison 2013) was performed on the predictive parameters and cumulative CO_2_ production. Prior to analysis, all variables were centered and scaled to unit variance. Due to missing C:N values, the parameter was excluded from the investigations.

## 3 Results

### 3.1 Sediment Properties

The sediment properties varied strongly among the samples (Table 1). TOC values ranged from 40.8 wt% in the surface peat of the DLB to 1.5 wt% in the cryopeg sediments of East Twin Lake. C:N and δ^13^C ratios varied strongest within the DLB and East Twin Lake core, respectively. The samples ranged in age from modern (UL10) to late Pleistocene age (42.57 cal ka BP in ETL252). With the exception of the surface samples from the upland and DLB (dominated by organic matter), samples were silty.

**TABLE 1:** Biogeochemical and hydrochemical conditions of the incubation samples.

Sample	Depth (cm)	Position	Sediment			^14^C (cal ka BP)	Water/ice			Color code
			TOC (wt%)	C:N	δ^13^C (‰ vs. VPDB)		Content (wt%)	EC (mS cm^−1^)	pH	
UL10	5–14	Active layer	21.6	16.6	−28.3	Modern	59.9	0.2^a^	5.4^a^	
UL75	70–80	Permafrost	20.0	19.5	−27.2	6.98 (±0.19)	74.4	0.6^a^	5.5^a^	
UL186	180–192	Permafrost	5.7	14.5	−26.9	13.26 (±0.08)	53.6	7.6^a^	7.8^a^	
WTL8	3–13	Talik	15.8	17.5	−28.6	2.28 (±0.16)	64.4	1.9	6.3	
ETL41	16–63	Cryopeg	14.0	17.5	−28.9	2.92 (±0.16)	56.8	14.6	6.1	
ETL215	208–221	Cryopeg	1.5	n.d.	−25.8	27.73 (±0.13)	18.0	38.8	7.4	
ETL252	249–254	Cryopeg	1.7	n.d.	−25.8	42.57 (±0.20)	18.5	39.6	7.6	
DLB15	10–19	Active layer	40.8	25.9	−26.8	0.68 (±0.05)^b^	94.8	2.1^a^	4.9^a^	
DLB31	26–35	Active layer	7.4	18.8	−27.8	1.27 (±0.03)	45.6	2.6^a^	5.5^a^	
DLB137	131–142	Refrozen saline permafrost	1.7	12.7	−25.0	34.13 (±0.25)	28.2	24.8^c^	8.0^c^	

*Note:* The sample name is the landform (UL—upland, WTL—West Twin Lake, ETL—East Twin Lake, DLB—Drained lake basin) plus the mean sample depth below sediment surface in cm. The color code on the right is the same as in Figures 2 and 3, as well as Table 2.

Abbreviation: n.d., not determined.

^a^ Average from sample above and below.

^b^ Age from sample below.

^c^ Value from sample above.

Active layer samples were characterized by high SWC, low EC, and acidic conditions (average pH 5.3). The permafrost domain showed intermediate ice contents, fresh to brackish ECs, and near‐neutral to slightly alkaline pH. Refrozen DLB permafrost sediments in comparison were saline and revealed a low TOC content (1.7 wt%). The talik and cryopeg sediments of the lakes were characterized by decreasing SWC and increasing EC as well as pH values with depth.

### 3.2 CO_2_ Production

All incubated samples produced CO_2_ without lag time after experiment start and CO_2_ production rates stabilized during the experiment (Figure S1). The strength of cumulative CO_2_ production was generally reflected in the production rates (Table S1). Maximum and minimum CO_2_ production occurred on average after 6.4 and 320.1 days, respectively.

#### 3.2.1 CO_2_ Production Normalized to Dry Weight

**FIGURE 2:**
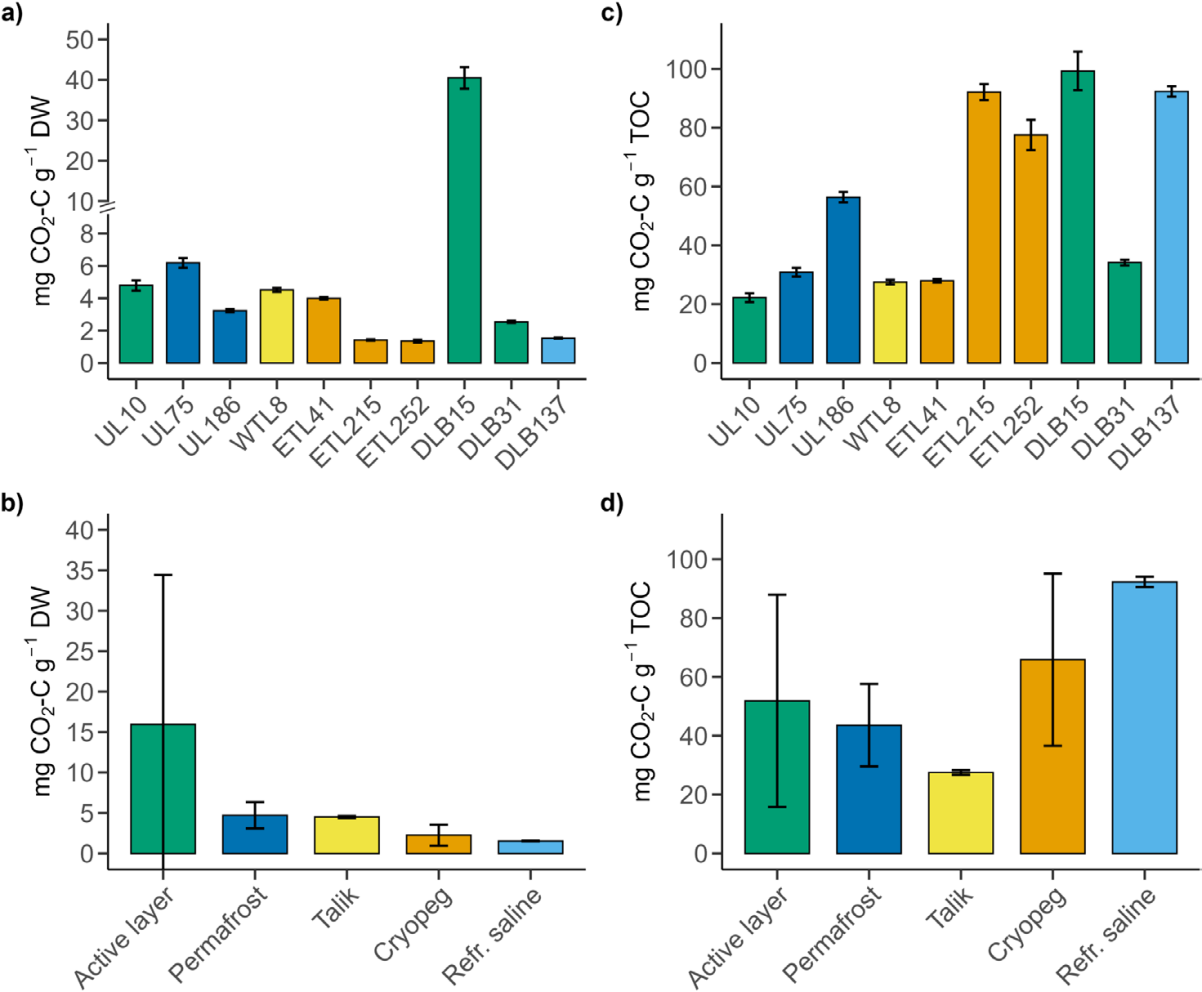
Cumulative carbon dioxide (CO_2_) production in milligram (mg), (a, b) per gram dry weight (DW), and (c, d) per gram total organic carbon (TOC) during the incubation experiment, including standard deviations (note the *y*‐axis break in panel a). In (a) and (c) individual incubation samples are visualized, which consist of three replicates (*n* = 3) each. The bar colors indicate in which sediment position samples are grouped in (b) and (d): Active layers (green, *n* = 9), permafrost (dark blue, *n* = 6), talik (yellow, *n* = 3), cryopeg (orange, *n* = 9), and refrozen saline permafrost (light blue, *n* = 3).

The average cumulative CO_2_ production per gram dry weight after 382 days was 7.0 ± 0.4 mg C g^−1^ DW (Figure 2a). DLB15 showed an order of magnitude higher CO_2_ production than any other site (40.5 ± 2.2 mg C g^−1^ DW). UL75 (6.2 ± 0.3 mg C g^−1^ DW), UL10 (4.8 ± 0.3 mg C g^−1^ DW), WTL8 (4.5 ± 0.1 mg C g^−1^ DW), and ETL41 (4.0 ± 0.1 mg C g^−1^ DW) produced moderate amounts of CO_2_. The lowest activity was recorded for UL186 (3.2 ± 0.1 mg C g^−1^ DW), DLB31 (2.5 ± 0.1 mg C g^−1^ DW), DLB137 (1.5 ± 0.0 mg C g^−1^ DW), ETL215 (1.4 ± 0.0 mg C g^−1^ DW), and ETL252 (1.4 ± 0.1 mg C g^−1^ DW). CO_2_ production decreased with sediment depth in the respective landforms (with the exception of UL75; Figure S1). A one‐way ANOVA on log‐transformed CO_2_ content revealed highly significant differences between samples (F_(9,20)_ = 1521, *p* < 0.001). Post hoc Tukey results showed that UL10‐WTL8 (*p* = 0.83), WTL8‐ETL41 (*p* = 0.07), ETL215‐ETL252 (*p* = 0.91), and ETL215‐DLB137 (*p* = 0.52) were sample pairs which were similar to another.

When samples were grouped by their position, active layer samples produced most CO_2_ (on average 15.9 ± 17.4 mg C g^−1^ DW), followed by the permafrost (4.7 ± 1.5 mg C g^−1^ DW) and talik (4.5 ± 0.1 mg C g^−1^ DW) domain. The cryopeg (2.3 ± 1.2 mg C g^−1^ DW) and refrozen saline permafrost (1.5 ± 0.02 mg C g^−1^ DW) samples produced least CO_2_ (Figure 2b). The one‐way ANOVA on log‐transformed data showed significant differences between positions concerning CO_2_ production (*F*_(4,25)_ = 4.58, *p* = 0.007). Since residuals deviated from normality, the non‐parametric Kruskal–Wallis test was applied to confirm the result, supporting significant differences among positions ($\chi_{\left(4\right)}^{2}$ = 14.55, *p* = 0.006). Thereby, the active layer differed significantly from the cryopeg (*p* = 0.01) and refrozen permafrost (*p* = 0.03).

#### 3.2.2 CO_2_ Production Normalized to TOC Contents

If normalized to TOC contents, 56.0 ± 2.3 mg C g^−1^ TOC were produced on average during the incubation (Figure 2c). DLB15 produced most CO_2_, with 99.3 ± 6.5 mg C g^−1^ TOC, followed by DLB137 (92.3 ± 1.7 mg C g^−1^ TOC) and ETL215 (92.1 ± 2.7 mg C g^−1^ TOC). ETL252 (77.5 ± 5.1 mg C g^−1^ TOC), UL186 (56.3 ± 1.8 mg C g^−1^ TOC), DLB31 (34.1 ± 0.9 mg C g^−1^ TOC), and UL75 (30.9 ± 1.5 mg C g^−1^ TOC) were characterized by moderate production. The lowest activity was recorded for ETL41 (27.9 ± 0.6 mg C g^−1^ TOC), WTL8 (27.5 ± 0.8 mg C g^−1^ TOC), and UL10 (22.2 ± 1.5 mg C g^−1^ TOC). No log‐transformation was needed for the one‐way ANOVA, and it indicated highly significant differences in CO_2_ among samples (*F*_(9,20)_ = 330, *p* < 0.001).

Grouped by position (Figure 2d), refrozen saline permafrost (92.3 ± 1.7 mg C g^−1^ TOC) and the cryopeg (65.9 ± 27.6 mg C g^−1^ TOC) samples produced most CO_2_, followed by the active layer samples (51.9 ± 34.0 mg C g^−1^ TOC). Permafrost (43.6 ± 12.8 mg C g^−1^ TOC) and talik (27.5 ± 0.6 mg C g^−1^ TOC) sediments produced least. The one‐way ANOVA indicated a modest but significant effect of position on CO_2_ production (*F*_(4,25)_ = 2.85, *p* = 0.045). However, Tukey HSD post hoc comparisons showed that none of the positions differed significantly from one another, indicating that CO_2_ normalized to TOC contents was statistically similar across active layer, permafrost, refrozen permafrost, talik, and cryopeg sediments. Only refrozen permafrost and the talik comparison approached significance (*p* = 0.0501).

### 3.3 *n*‐Alkane Patterns

The general *n*‐alkane patterns showed that TAC increased strongly while the ACL remained relatively stable and CPI increased moderately (Table 2). Nevertheless, within TAC values, large differences exist. DLB137 was relatively alkane depleted, which remained unchanged after the incubation experiment and TAC had strongly increased in DLB15 after one year. Both before and after the incubation, TAC was highest in ETL41. When calculated in μg g^−1^ TOC, the general picture was similar (Table S2).

**TABLE 2:** Total *n*‐alkane content (TAC), average chain length (ACL), and carbon preference index (CPI) before (pre) and after (post) the incubation experiment of 382 days.

Sample	TAC			ACL			CPI			Color code
	Pre (μg g^−1^ DW)	Post (μg g^−1^ DW)	Change (%)	Pre	Post	Change (%)	Pre	Post	Change (%)	
UL10	113.3	222.1	96	26.2	26.2	0	10.6	11.4	8	
UL75	35.9	148.3	313	27.1	26.9	−1	21.5	21.7	1	
UL186	5.1	11.2	120	27.6	27.0	−2	15.6	16.2	4	
WTL8	192.2	600.5	212	25.5	25.1	−2	6.9	7.5	9	
ETL41	305.4	949.9	211	25.3	25.2	0	6.5	6.7	3	
ETL215	1.9	3.4	79	26.2	25.5	−3	5.1	6.7	31	
ETL252	17.0	10.1	−41	26.4	26.1	−1	7.0	7.7	10	
DLB15	12.8	80.6	530	27.5	27.8	1	37.2	62.5	68	
DLB31	107.8	121.2	12	25.6	25.7	0	8.9	7.5	−16	
DLB137	1.5	1.5	0	28.2	26.5	−6	12.5	10.5	−16	
Average	79.3	214.9	153	26.6	26.2	−1	13.2	15.8	10	

*Note:* The color code on the right is the same as in Figures 2 and 3, as well as Table 1.

Since ACL values remained relatively unchanged, alkane homologues changed relatively homogeneously. However, there was a tendency towards shortening chain lengths. This picture was not reflected in the CPI index. It indicated that the CPI increased on average. Yet, relatively large differences occurred along samples. DLB15 showed the strongest increase of CPI while DLB137 indicated the strongest CPI decrease.

### 3.4 Microbial Abundances

**FIGURE 3:**
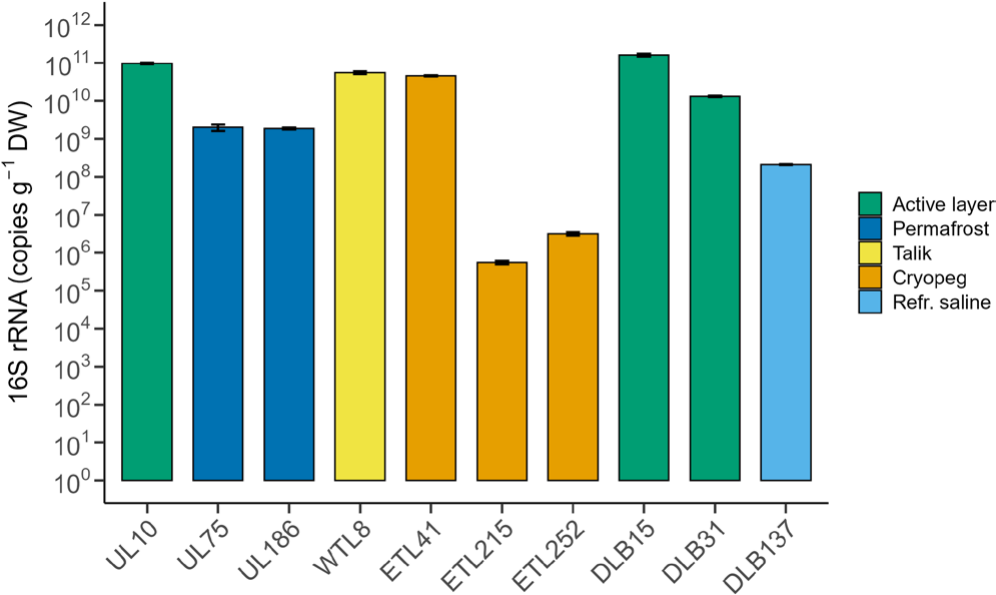
Bacterial abundance based on 16S rRNA gene copy numbers including standard deviations at day 0 of the incubation experiment. Individual samples consist of three replicates and are colored according to their position (see legend).

16S rRNA abundances varied substantially, ranging from 5.5 × 10^5^ copies g^−1^ DW (ETL215) to 1.6 × 10^11^ copies g^−1^ DW (DLB15) in individual samples (Figure 3). Grouped after position, active layer samples had the highest abundances (9.1 × 10^10^ copies g^−1^ DW), followed by the talik (5.5 × 10^10^ copies g^−1^ DW), the cryopeg (1.5 × 10^10^ copies g^−1^ DW), permafrost (1.93 × 10^9^ copies g^−1^ DW), and refrozen saline permafrost (2.1 × 10^8^ copies g^−1^ DW) samples.

### 3.5 Relations Between CO_2_ Production and Predictive Parameters

**FIGURE 4:**
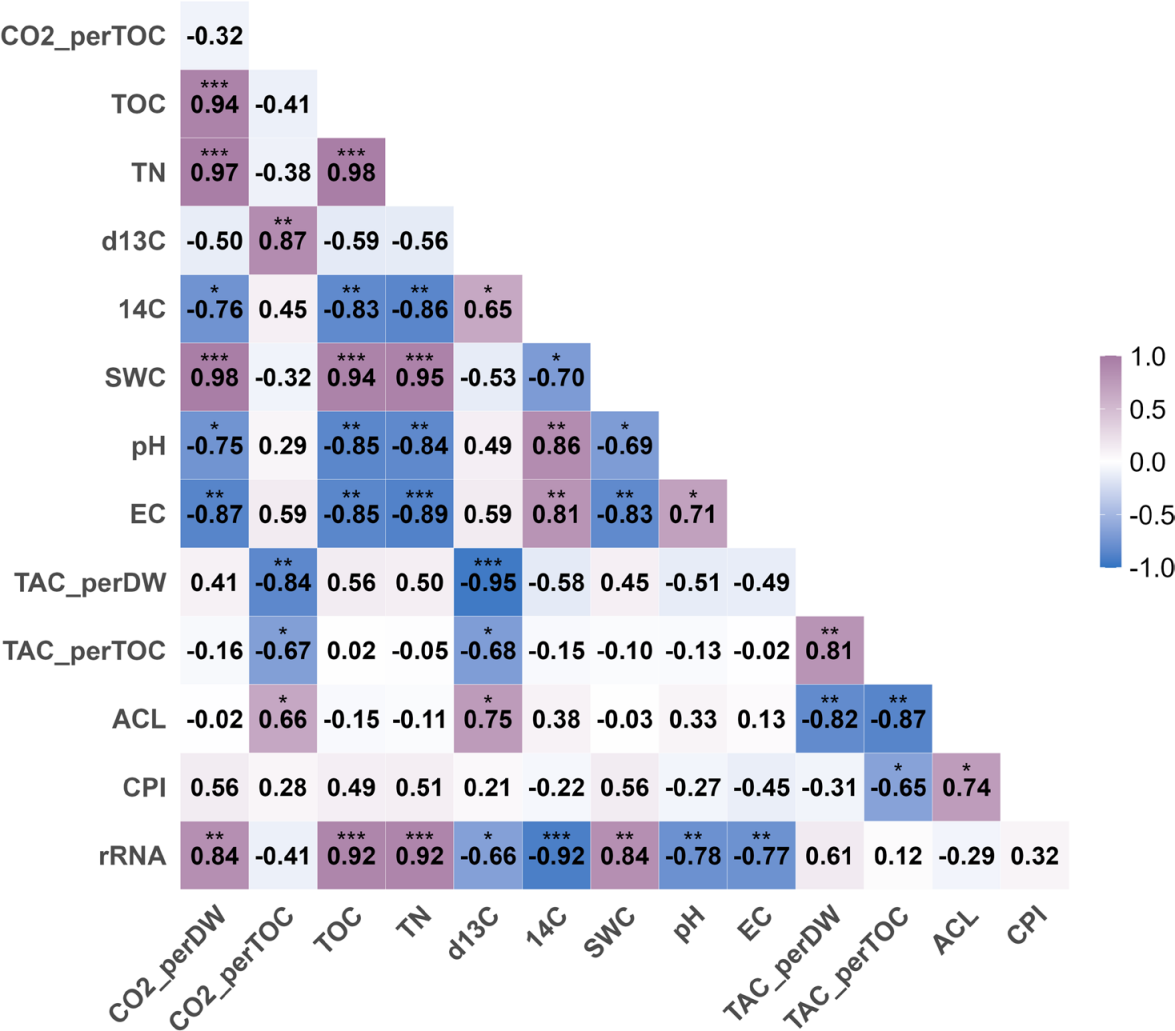
Matrix illustrating Spearman correlation coefficients (*ρ*, positive values in purple, negative values in blue) and *p*‐values (* < 0.05, significant; ** < 0.01, very significant; *** < 0.001, highly significant) of cumulative CO_2_ production (after the incubation length of 382 days) and predictive parameters. CO_2_ production is normalized to dry weight (CO2_perDW) and total organic carbon (CO2_perTOC). Parameters include: TOC (total organic carbon), TN (total nitrogen), δ^13^C (stable carbon isotope ratio), 14C (radiocarbon date), SWC (soil water content), pH, EC (electrical conductivity), TAC_perDW (total *n*‐alkane content per gram sediment dry weight), TAC_perTOC (total *n*‐alkane content per gram total organic carbon (TOC)), ACL (*n*‐alkane average chain length), CPI (*n*‐alkane carbon preference index), rRNA (16S rRNA microbial abundance).

Parameters significantly correlating with the cumulative CO_2_ production per gram dry weight are TOC (*ρ* = 0.94, *p* < 0.01), TN (*ρ* = 0.97, *p* < 0.01), ^14^C (*ρ* = −0.76, *p* = 0.01), SWC (*ρ* = 0.98, *p* < 0.01), pH (*ρ* = −0.75, *p* = 0.01), EC (*ρ* = −0.87, *p* < 0.01), and 16 rRNA (*ρ* = 0.84, *p* < 0.01). Repeated correlation tests with cumulative CO_2_ production per gram TOC showed that δ^13^C (*ρ* = 0.87, *p* < 0.01), TAC per g^−1^ DW (*ρ* = −0.84, *p* < 0.01), TAC per g^−1^ TOC (*ρ* = −0.67, *p* = 0.03), and the ACL (*ρ* = 0.66, *p* = 0.04) build significant pairs. A correlation matrix is visualized in Figure 4.

**FIGURE 5:**
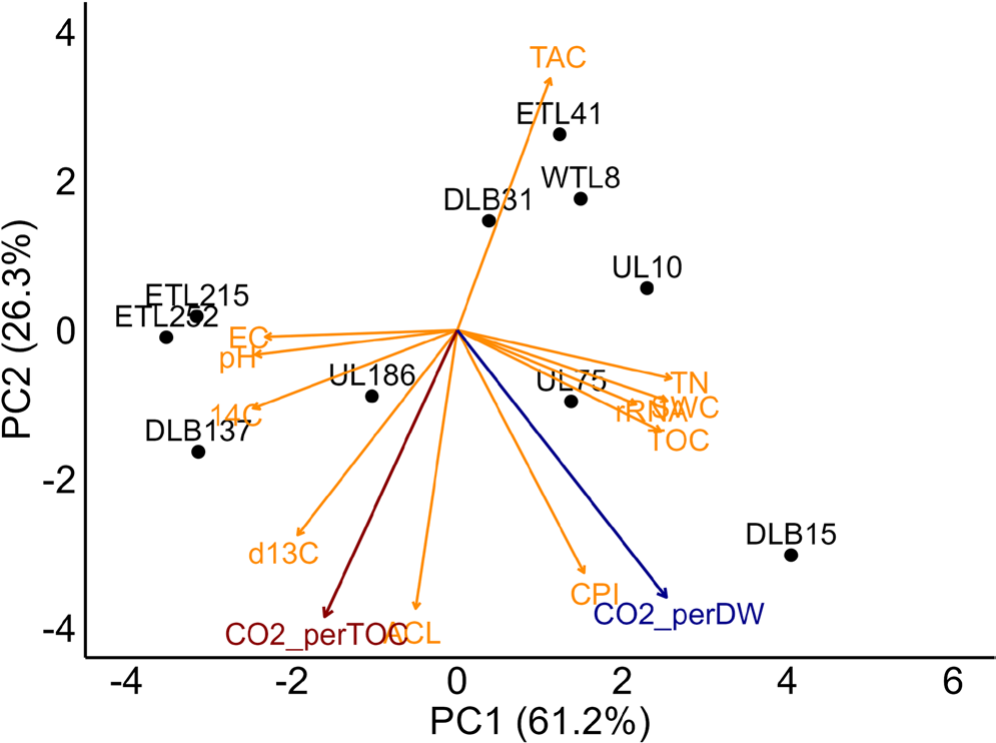
PCA biplot of incubation samples with vectors of predictive parameters (yellow) and cumulative CO_2_ (in blue for normalization after DW, in red for normalization after TOC). The overlapping predictive parameter vectors (yellow) are those of SWC and 16S rRNA.

In the PCA, principal component (PC) 1 explained 61.2% of the variance in the data while PC2 accounted for 26.3% (Figure 5). Deep samples (ETL215, ETL252, DLB137, UL186) were associated with relatively alkaline pH values, high ECs, older ^14^C‐ages, and heavier δ^13^C values. Lake sediments (WTL8, ETL41) and active layers (UL10, DLB31) were characterized by high alkane contents. UL75 was unique due to its relatively high CPI value. DLB15 was an outlier due to its generally strong CO_2_ production along with the highest TOC, TN, and SWC, CPI values, and microbial abundance. If CO_2_ production normalized to TOC was plotted in the PCA, the CO_2_ vector changed more towards the direction of the deep samples (DLB137, ETL215, ETL252, UL186).

## 4 Discussion

### 4.1 CO_2_ Production in Arctic Coastal Plain Sediments

Comparing CO_2_ production normalized to dry weight and total organic carbon provides insights into the metabolic potential of the respective samples. Under in situ conditions (i.e., normalization to DW), active layers produced most CO_2_ (Figure 2b), a pattern also found at other study sites (Baysinger et al. 2025). However, when CO_2_ production was expressed on a TOC basis, a very different pattern emerged: refrozen saline permafrost and the cryopeg produced more CO_2_ than the active layer, although differences were not significant (Figure 2d). This stresses that potentially occurring TOC‐rich saline permafrost and unfrozen‐cryotic sediments hold strong CO_2_ production potentials. ETL41 (upper cryopeg sediment) hints towards such a scenario as the sample has an elevated TOC content (14 wt%) as well as brackish porewater (Table 1), leading to the strongest CO_2_ production of the three cryopeg samples when normalized to dry weight. Organic‐rich and saline sediments may generally be found where saline porewater migrates upwards from underlying marine deposits due to bottom‐up freeze‐back of taliks (Mackay 1997; Stephani et al. 2020). Further, organic matter may receive salts from periodic inundation or sea spray in coastal settings (Funk et al. 2004). The spatial extent and characteristics of the saline permafrost region remains poorly constrained, calling for mapping efforts, which should particularly include cryopegs as well.

TOC‐normalization provided the insight into the potential CO_2_ production of saline sediments, as this approach represents a scenario in which all samples contain equal amounts of organic carbon, highlighting the intrinsic degradability of the carbon pool rather than its abundance. While refrozen saline permafrost conditions were represented by one composite sample (three laboratory replicates), the cryopeg and the active layer were represented each by three samples (nine replicates), lending greater confidence to these observed results. Tanski et al. (2021) reported a similar shift in observed CO_2_ production depending on the normalization method (i.e., normalization to DW vs. TOC content), underscoring the importance of considering both approaches in GHG assessments. Detailed discussions on CO_2_ productions in different sediment types and driving factors follow below.

#### 4.1.1 Active Layers

When focusing on the active layer of the upland and the DLB, large differences in CO_2_ production occurred, causing a high standard deviation (Figure 2b). While UL10 (4.8 ± 0.3 mg C g^−1^ DW) and DLB31 (2.5 ± 0.1 mg C g^−1^ DW) produced moderate amounts of CO_2_, production was significantly higher in DLB15 (40.5 ± 2.2 mg C g^−1^ DW; Figure 2a). This comparison shows, as also observed by Faucherre et al. (2018), that sediment type is the determining factor for CO_2_ production rather than the active layer per se. Our chosen active layer samples therefore exemplify that a diverse set of samples is needed to simulate the range of CO_2_ production in seasonal thaw layers. Due to their landscape position, DLBs are generally wetter than other surrounding landscape units, which limits oxygen availability and reduces decomposition rates, thereby favoring organic matter sequestration (Hinkel et al. 2003; Jones et al. 2012). As active layer depth might exceed the surface peat thickness though (Bockheim et al. 2004), focusing purely on surface sediments would distort the study of active layer CO_2_ production potentials. The observed CO_2_ patterns in the active layers were still supported when normalizing CO_2_ production to TOC (Figure 2c,d), similarly to data reported by Herbst et al. (2024).

DLB15 is a peat sample because of the above‐described process that occurred since lake drainage about 700 cal a BP (Table 1; Seemann et al. 2026). This characterized the sample with the highest TOC and TN contents, highest organic carbon quality (C:N, CPI), highest SWC, most acidic pH, and strongest microbial abundance. Considering the PCA, DLB15 together with its parameters therefore represents the end‐member of all incubation samples (Figure 5). Although C:N could not be statistically tested against cumulative CO_2_ production in the Spearman analysis due to missing values (Table 1), and CPI values revealed no significant correlation, Tanski et al. (2021) proved both quality indexes as predictive parameters for CO_2_ production.

#### 4.1.2 Permafrost and Refrozen Permafrost

The permafrost domain as represented by UL75 (6.2 ± 0.2 mg C g^−1^ DW) and UL186 (3.2 ± 0.1 mg C g^−1^ DW) showed that samples closer to the surface have a stronger potential to produce CO_2_ upon thaw (Figure 2a). This observation is important because those depths are at risk of near‐future seasonal thaw and subsequent GHG production, due to active layer deepening (Liu et al. 2024).

DLB137 (1.5 ± 0.0 mg C g^−1^ DW) by definition is a permafrost layer, too, as permafrost re‐aggraded since the former lake drained, a process usually happening within very short times after drainage on the Alaskan North Slope (Ling and Zhang 2004). This however means that these sediments were thawed during the lake phase, which potentially lowered the carbon availability (i.e., labile carbon) and its quality (C:N, Table 1) leading to a smaller CO_2_ production nowadays when compared to the upland samples. Per gram TOC however, the CO_2_ production potential was similar to DLB15 (Figure 2c) indicating that DLB permafrost behaves differently than upland permafrost. An influencing factor for the pattern of CO_2_ production in the DLB137 sample is likely a marine signature as indicated by the saline pore water (Table 1). Even though the bulk radiocarbon age of the sample (34.13 ± 0.25 cal ka BP; Seemann et al. 2026) places it in the late Pleistocene, that is, after the most recent phases of marine deposition in the study area (Section 2.1), the marine influence can be explained through upward migration of saline porewater from underlying marine sediments when the former talik started to refreeze upwards, pushing and concentrating saline porewater upwards until complete freeze‐back (Mackay 1997; Stephani et al. 2020).

In similar aged Yedoma deposits, Knoblauch et al. (2013) quantified a CO_2_ production of 1.6 mg C g^−1^ DW. Although the incubation ran for 1200 days at 4°C aerobically, this comparison indicates a comparable CO_2_ response to thawing. Another likely reason for the low CO_2_ production is the comparably lower microbial abundance based on 16S rRNA gene copy numbers (Figure 3). Such a pattern of low microbial abundances in Pleistocene sediments was also observed by Knoblauch et al. (2021).

#### 4.1.3 Talik and Cryopeg

Although lake sediments exhibit anaerobic conditions, we conducted aerobic incubations due to potential future drainage and coastal erosion events which would expose these sediments to oxygen (Nitze et al. 2017; Tanski et al. 2019). The surface samples of the talik (WTL8) and cryopeg (ETL41) were in the range of permafrost samples (UL10, UL186) and had greater potential compared to the deeper cryopeg sediments (DW normalized, Figure 2a). This is likely attributed to a larger TOC availability (Table 1), which is also reflected in the *n*‐alkane content (Table 2). Also, the microbial abundance was higher than in samples below (Figure 3).

Even if unfrozen sediments produce less CO_2_ than active layers, CO_2_ in deep taliks may migrate through the unfrozen sediment, remaining relevant for potential emissions (Pellerin et al. 2022). This is especially relevant for cryopegs for two reasons: first, they are potential CO_2_ hotspots, as discussed earlier; and second, production can occur already at sub‐zero temperatures due to their unfrozen state in the field. Studies on the biogeochemistry and microbiology of cryopegs are generally limited (Gilichinsky et al. 2005; Iwahana et al. 2021; Rapp et al. 2021) and to our knowledge GHG production has previously not been investigated in these sediments. Future studies should focus on microbial dynamics and carbon mineralization in saline deposits and cryopegs under various environmental conditions (e.g., anaerobic). This is crucial due to the potentially large pan‐Arctic abundance, leading to faster‐than‐anticipated landscape changes with consequences for the permafrost carbon budget (Brouchkov 2003; Seemann et al. 2026).

### 4.2 Patterns and Drivers of Aerobic CO_2_ Production

Our observed GHG productions lie well within ranges quantified in other experiments (Baysinger et al. 2025; Herbst et al. 2024; Lee et al. 2012), which confirms the relevance of saline refrozen and cryopeg sediments. For example, Lee et al. (2012) found both lower (1.1 mg C g^−1^ DW; 15.7 mg C g^−1^ TOC) and higher (71.9 mg C g^−1^ DW; 182.1 mg C g^−1^ TOC) CO_2_ production rates in mineral and organic sediments from Alaska, respectively. Their incubation was conducted aerobically for 500 days at 15°C. As the GHG production in organic soils may be multiple times larger than in mineral soils (Lee et al. 2012), DLB15 is a representative outlier to our study.

CO_2_ production peaked early in the incubation (around day 6) and decreased to minimal rates by day 320, indicating that the conducted 382‐day incubation captured the initial and intermediate phases of carbon mineralization. For assessments of the long‐term fate of organic carbon, longer experiments are required though as demonstrated by Knoblauch et al. (2018) for example. The observed temporal pattern of CO_2_ production with strong initial production and stabilizing production after first response is commonly observed (Dutta et al. 2006; Walz et al. 2017; Wickland and Neff 2008). This can be explained by the reactivity of carbon pools, after which labile carbon is mineralized quickly while the stable pool decomposed over longer periods (Dutta et al. 2006; Knoblauch et al. 2013; Schädel et al. 2013). From a microbiological point of view, microbial activity peaks after thaw, stabilizes and slowly declines over time unless environmental changes occur (Jenrich et al. 2024; Waldrop et al. 2021).

The results of the incubation under specific, controlled and isolated conditions cannot be directly translated to the field, for example because soil dynamics (e.g., litter input, temperature variability) were not simulated in this approach. Such processes could substantially alter mineralization rates, leading both to increasing or decreasing productions. Instead, the incubation rather informs process understanding under set conditions, which can be used to assess carbon mineralization potentials of different sediment types. The incubation temperature of 10°C, as approximately representative of the maximum average daily air temperature in Utqiaġvik (Alaska Climate Research Center 2026), thereby simulates the potential peak carbon mineralization. Other studies focus for instance on broader seasonal conditions, like the open‐water season, to assess potential GHG release from eroding coastal cliffs (Tanski et al. 2019).

The large variability among sediment properties (Table 1) provided the possibility to assess the driving factors of CO_2_ production. Correlation tests revealed that TOC and TN correlate with the cumulative CO_2_ production, as observed in other Arctic sediments (Baysinger et al. 2025; Dutta et al. 2006; Knoblauch et al. 2013, 2021). Furthermore, we found a negative correlation between CO_2_ production (DW basis) and ^14^C ages indicating that younger deposits have a larger CO_2_ production potential. This likely results from the negative correlation between sediment age and TOC content (Figure 4). However, this pattern is not universal, as, for example, Yedoma sediments typically do not exhibit a TOC age‐depth trend (Jongejans et al. 2018). Although older sediments are found in relatively deeper deposits (Table 1), these sediments remain relevant for potential GHG emissions as abrupt thaw processes can lead to permafrost degradation of deep sediments (Walter Anthony et al. 2018; Webb et al. 2025). Also, this pattern is offset if production is normalized to TOC, pointing to the relevance of potentially old and carbon‐rich sediments.

Next to sedimentological parameters, we also found that the hydrochemical parameters (SWC, pH, and EC) are key factors of CO_2_ production. Baysinger et al. (2025) tested that these dominate over TOC as a predictive factor, which is also true for the SWC in our case (Figure 4). Yet we argue that the correlation with cumulative CO_2_ production is at least partly the result of the relation between sediment type and SWC. The SWC is calculated as the percentage weight difference between wet and dry weight, which results in typically higher contents in lighter surface peat compared to heavier mineral soil. Thus, caution has to be taken when scaling findings on the role of SWC to landscape level. This is supported by Wickland and Neff (2008) who found that neither low nor high moisture (leading to anaerobic conditions) levels promote carbon mineralization, but rather intermediate levels (~25%–75%).

Extreme pH values are known to impact microbial abundance and activity and with that GHG production (Hodgkins et al. 2014; Song et al. 2020). As we found a negative correlation between pH and CO_2_ production (i.e., strong production in acidic conditions), but also a negative correlation between pH and TOC content (Figure 4), the TOC content likely remains the dominant driver of produced CO_2_. pH values could not be re‐measured after the incubation due to inefficient amounts of pore water, but it can be expected that after thawing—when labile carbon became available—pH values increased (Hodgkins et al. 2014).

The negative correlation between EC and cumulative CO_2_ (DW basis) classified salinity as an inhibiting production factor. However, this result contradicts the finding of Tanski et al. (2019) that CO_2_ production in seawater increases once organic matter erodes into the near‐shore zone. They argue that saltwater may facilitate labile carbon mobilization, as flocculation in seawater preferentially removes less labile fractions (Dou et al. 2008). Since the positive correlation between CO_2_ normalized to TOC and EC was not significant (Figure 4), the TOC content once again likely remains the determining factor for CO_2_ production.

Correlation tests revealed a significant correlation between 16S rRNA gene copy numbers and cumulatively produced CO_2_, confirming heterotrophs as responsible for CO_2_ production under aerobic conditions (Knoblauch et al. 2021).

Overall, TOC content appears to be the most important parameter for CO_2_ production. When normalized with TOC contents, in order to shift the perspective away from carbon quantity, CO_2_ production revealed correlations with δ^13^C, alkane content and ACL (Figure 4). This indicates that beyond the total amount of organic carbon, its characteristics (e.g., lability and source) also act as key factors for the mineralization potential, as those parameters are established quality proxies (Strauss et al. 2015). Source and degradation signals of δ^13^C are generally difficult to distinguish but as our samples all clearly indicate terrestrial or lacustrine origins (Meyers 1994), we interpret the positive correlation with cumulative CO_2_ as the degradation signal. The role of the *n*‐alkane proxies in light of the incubation experiment are discussed below (Section 4.3). Lastly, we could not conduct statistical tests with C:N due to missing values. However, because both TOC and TN correlated with cumulative CO_2_ (DW basis), it is reasonable to expect that C:N, as a degradation proxy, also plays an important here, like previously described by Schädel et al. (2014).

### 4.3 Alteration of *n*‐Alkane Patterns

As organic carbon quantity and quality are drivers of CO_2_ production, carbon investigations on the molecular level enrich the understanding of the permafrost carbon cycle. On the one hand, *n*‐alkane biomarker analyses have been conducted prior to incubation experiments to assess GHG production potentials (Jongejans et al. 2021; Yang et al. 2023). On the other hand, however, no repeated analyses after incubations have yet been conducted to trace biochemical changes throughout organic carbon mobilization and mineralization in a permafrost context.

We observed a strong variation in the initial *n*‐alkane content (1.5–305.4 μg g^−1^ DW), while post‐incubation TAC revealed even stronger differences (1.5–949.9 μg g^−1^ DW; Table 2). On average, TACs increased by 153%, though the range was large (−41% to +530%). TAC normalization to TOC contents showed similar patterns (Table S2). Interestingly, TAC (normalized to DW and TOC) did not correlate with cumulative CO_2_ production on a dry weight basis, but significant negative correlations appeared when CO_2_ was normalized to TOC (Figure 4). This raises two questions: (i) why did TAC increase in most samples during the incubation, and (ii) why does TAC correlate negatively with CO_2_ production (normalized to TOC)?

Concerning question i: under microbial degradation, a decline in TAC (as organic compounds) could be expected, as demonstrated by Li et al. (2017). This pattern was indeed observed in ETL252, but not in DLB137 (net zero change) and all other samples (TAC increases). One possible explanation is the production of alkanes by microbial and fungal taxa. Because microbes and fungi can produce similar *n*‐alkane patterns (Zech et al. 2011), it is not possible to distinguish between their respective contributions in our study. Bacteria can synthesize short‐chain *n*‐alkanes (< nC22), typically with CPI values around 1 (Killops and Killops 2005; Tipple and Pagani 2010). However, bacterial and fungal enzymes can also generate long‐chain alkanes via fatty acid decarboxylation and reduction (Jansen and Nierop 2009; Ladygina et al. 2006; Rojo 2009). Since Spearman tests revealed no significant correlation between TACs and microbial abundance (Figure 4), and newly produced microbial/fungal alkanes would be expected to reduce CPI rather than increase it, as observed in our case, we consider their contribution to TAC increases as minor.

A second explanation is algal production of alkanes. Algal *n*‐alkane distributions can resemble those of higher plants, with long‐chain homologues and pronounced odd‐over‐even predominances (J. G. Jones 1969). This distinguishes them from microbial/fungal sources and could explain why TAC and also CPI increased. We hypothesize that algal inputs contributed especially in the lake sediments (WTL8, ETL41) where the greatest alkane contents occurred, despite moderate CO_2_ production.

Finally, an apparent increase in TAC following the incubation may result from preferential loss of labile organic fractions (Schmidt et al. 2011) and desorption of mineral‐associated lipids (Jong et al. 2024; Martens et al. 2023). Microbial mineralization of labile carbon to CO_2_ thereby reduces the denominator of the organic pool and leaves relatively recalcitrant *n*‐alkanes enriched; simultaneously, the incubation at 10°C and under aerobic conditions mobilized previously sorbed alkanes, increasing their extractable concentration. This would keep the ACL stable, since the carbon source remains the same, and the latter process would give another possible explanation for the apparent increase in the CPI.

The absence of a notable ACL change despite TAC and CPI changes, confirms ACL as a valuable source proxy in paleoenvironmental studies. However, the increase in TAC and CPI after thaw raises questions about the reliability of these well‐established environmental proxies. Multi‐year experiments (also under anaerobic conditions) would be interesting, to observe how these proxies continue to evolve over time (i.e., whether the increase in CPI is a short‐term effect which ceases once the labile carbon pool is exhausted).

Concerning question ii about the negative relation between TAC and CO_2_ production (normalized to TOC): We see that least alkane‐rich and alkane‐producing sediments revealed strongest CO_2_ productions (ETL215, ETL252, DLB137) causing the negative relation. This might be an indication that the alkane pool was either always relatively small in these samples, or that the alkane pool is relatively exhausted/mineralized. Thus, CO_2_ production occurs via different carbon pools in these samples, thereby contributing to the negative relation between TAC and CO_2_ production.

Overall, our findings suggest multiple, potentially interacting processes encompassing limited microbial/fungal alkane biosynthesis, algal contributions, preferential preservation and desorption processes of long‐chain alkanes. As our pre‐ and post‐incubation biomarker results do not conclusively resolve these pathways, future studies should aim to elucidate them in greater detail, informing the molecular carbon cycle.

### 4.4 Implications of Observed CO_2_ Production for Arctic Coastal Plains

Even though past marine transgressions resulted in the widespread presence of saline sediments in Arctic coastal plains (Brouchkov 2003; Hinkel et al. 2005), the relevance of saline permafrost and cryopegs for the permafrost carbon balance remains poorly studied and is therefore unaccounted for in current GHG assessments (Hugelius et al. 2024; Ramage et al. 2024; Schuur et al. 2022; Vonk et al. 2025). Our results highlight that carbon dynamics in Arctic lowlands are shaped not only by saline deposits but also by the characteristics of surface peats. These organic‐rich deposits are dominantly found in wetland tundra, which often cover more than 50% of Arctic coastal lowlands (Jones et al. 2022), underscoring the importance of potential aerobic exposure through drying for example. Although wetlands are overall CO_2_ sinks (Ramage et al. 2024), residual ponds following lake drainage can act as CO_2_ hotspots (Loiko et al. 2026). Continued drainage of basin ponds, as observed near Utqiaġvik (Andresen and Lougheed 2015), may further promote aerobic carbon mineralization through the exposure of sediments, thereby weakening the CO_2_ sink function of DLB wetlands. These processes are particularly relevant in regions exhibiting thermokarst lake drainage trends, such as the Yamal Peninsula and the northern Seward Peninsula (Nitze et al. 2018). But even in regions with net‐zero lake change, like the Yukon Delta or our study region (Nitze et al. 2018), the dynamic nature of thermokarst lake formation and drainage repeatedly lead to periodic exposure of lake sediments. With ongoing climate warming, lake taliks are increasingly at risk to incompletely refreeze following drainage (Jones et al. 2022), thereby prolonging thawed conditions and sustaining GHG production and emissions from those areas.

Subsurface saline and cryopeg sediments exhibited high TOC‐normalized CO_2_ potentials, despite showing low absolute CO_2_ production, indicating that potentially carbon‐rich saline deposits may be highly reactive. This aligns with anaerobic incubations by Dolle et al. (2025), who found higher CO_2_ production in in situ saline sediments compared to non‐saline ones. Further, Dolle et al. (2025) demonstrated that seawater inundation can enhance CO_2_ production in non‐saline permafrost up to 3.5 times. Along the land–ocean continuum, thermokarst lagoons similarly show elevated CO_2_ potentials (Jenrich et al. 2024, 2025), especially in newly formed lagoons. Combined with the fact that saline sediments can sustain microbial activity at sub‐zero temperatures due to freezing‐point depression, these findings emphasize the vulnerability of saline permafrost and cryopegs to climate change.

At coastal cliffs, active layers, saline permafrost, taliks, and cryopeg sediments are exposed and thawed, initiating renewed erosion. The timing of CO_2_ production is crucial here, as peak production occurred after only six days in our incubations. Given that unconsolidated coastal permafrost cliffs can reach heights of up to 40 m (Irrgang et al. 2022) and erode several meters per year (Jones et al. 2018), with rates expected to increase under continued warming (Nielsen et al. 2022), these cliffs may represent intense CO_2_ sources, complementing their established role in supplying particulate and dissolved organic carbon to nearshore waters (Nielsen et al. 2024; Ramage et al. 2024; Vonk et al. 2025). Once eroded into coastal waters, mineralization of dissolved and particulate organic carbon can further accelerate, as shown by Tanski et al. (2019) and Tanski et al. (2021).

A back‐of‐the‐envelope calculation illustrates how the effect of salinity may be able to substantially alter the CO_2_ balance of coastal permafrost regions. We focused on three representative landscape units from Ramage et al. (2024): dry tundra (uplands), wetland tundra (DLBs), and lowland abrupt thaw lakes (WTL, ETL). Under present conditions, these units collectively form a modest CO_2_ source of roughly 2 g C m^−2^ yr.^−1^ when normalized by their relative areal distribution in the pan‐Arctic (87% dry tundra, 6% wet tundra, 7% lakes; Ramage et al. 2024). Introducing salinity driven carbon mineralization, however, may change this balance. Based on our TOC‐normalized incubation, the salinity could (i) increase CO_2_ production by 57% in tundra uplands (production difference between UL10/UL75/UL186 and UL10/DLB137), (ii) dampen the CO_2_ sink function of wetland tundra by 36% (shift from DLB15/DLB31/UL75/UL186 production to DLB15/DLB31/DLB137 production), and (iii) increase CO_2_ production by 140% in lake cryopegs in comparison to lake taliks. Applying these factors to the mean CO_2_ fluxes reported by Ramage et al. (2024), the combined flux of the three units increases from 11.8 Tg CO_2_–C yr.^−1^ to approximately 30.6 Tg CO_2_–C yr.^−1^. Expressed per unit area, this shift corresponds to an increase from ~2 to ~5 g C m^−2^ yr.^−1^ for the pan‐Arctic distribution of those landscapes. But because Arctic coastal lowlands are typically a more lake and wetland dominated mosaic (assuming 30% dry tundra, 50% wet tundra, 20% lakes based on Jones et al. 2022), the areal normalized CO_2_ flux increases to nearly 11 g C m^−2^ yr.^−1^. This would result in a more than 5‐fold amplification relative to the status quo. Although upscaling our incubation results represents a highly speculative scenario, it demonstrates that salinity has the potential to substantially alter CO_2_ balances of Arctic coastal lowlands and thus calls for consideration in permafrost carbon assessments.

## 5 Conclusions

Our study demonstrates that the Alaskan Arctic Coastal Plain exhibits a broad range of CO_2_ production potentials. Carbon‐rich active layer sediments produced most CO_2_, whereas carbon‐poor lake cryopeg deposits and refrozen saline DLB permafrost exhibited low production after 382 days of incubation. However, when normalized to organic carbon content, refrozen saline permafrost and cryopeg sediments displayed similar production potentials as the active layer. This identifies potentially carbon‐rich saline deposits as key constituents of the changing permafrost carbon cycle.

The magnitude of CO_2_ production was primarily controlled by TOC contents but was also significantly influenced by sedimentary characteristics like age and nitrogen content, hydrochemical conditions (SWC, pH, EC), and microbial abundance. The carbon characteristics (δ^13^C, alkane content, ACL, and likely C:N) were found to be of significant meaning when production rates were normalized to TOC. Translating these laboratory findings to in situ conditions highlights the importance of biochemical and thermal settings, especially in cryopeg systems as they facilitate year‐round GHG production under unfrozen cryotic conditions.

Repeated biomarker analyses revealed that *n*‐alkane biomarkers are a valuable tool for gaining molecular insights into carbon cycle processes in permafrost regions. Their response to organic carbon mineralization was unexpected and should be a focus in future studies. An overall increase in alkane content, accompanied by an average CPI increase, can likely be explained by newly produced alkanes, preferential degradation of labile carbon, and desorption processes.

This study provides a first step toward understanding biochemical responses of so far neglected terrestrial saline permafrost and cryopeg deposits to ongoing climate change. As saline deposits are found in the extensive circum‐Arctic coastal regions, our findings underscore the need for future research to further constrain the role of saline permafrost systems in the regional and global carbon cycle. We underlined this need with a first‐order upscaling based on our incubation results combined with literature values, which suggest that salinity may substantially modify CO_2_ budgets in Arctic coastal lowlands, with the potential to locally amplify CO_2_ fluxes by up to 5‐fold.

## Author Contributions

**Fabian Seemann:** conceptualization, investigation, funding acquisition, writing – original draft, methodology, visualization, writing – review and editing, formal analysis, data curation. **Mackenzie R. Baysinger:** writing – review and editing, methodology, data curation, formal analysis. **Susanne Liebner:** resources, supervision, writing – review and editing, methodology. **Claire Treat:** methodology, writing – review and editing, supervision, conceptualization. **Michael Zech:** writing – review and editing, supervision, resources. **Maren Jenrich:** conceptualization, methodology, writing – review and editing, funding acquisition. **Guido Grosse:** conceptualization, writing – review and editing, resources, supervision, funding acquisition. **Benjamin M. Jones:** conceptualization, writing – review and editing, resources, funding acquisition. **Jens Strauss:** conceptualization, writing – review and editing, methodology, supervision, resources, funding acquisition.

## Funding

This work was supported by the National Science Foundation (1806213, 2336164), Alfred Wegener Institute Helmholtz Centre for Polar and Marine Research, and Deutsche Bundesstiftung Umwelt.

## Conflicts of Interest

The authors declare no conflicts of interest.

## Supporting information


**Figure S1:** CO_2_ production per gram dry weight (DW) and per gram total organic carbon (TOC) during the incubation experiment, including standard deviation (shaded areas). (a) UL samples, (b) West Twin Lake (WTL) and East Twin Lake (ETL) samples, (c) DLB samples (note the *y*‐axis break of the left panel).
**Table S1:** CO_2_ production rates during 382 days of aerobic incubation, and days at which extreme values occurred.
**Table S2:** Total *n*‐alkane content (TAC in μg g^−1^ TOC) before and after the aerobic incubation experiment of 382 days.

## Data Availability

Supporting data is freely accessible from the PANGAEA open access archive: https://doi.pangaea.de/10.1594/PANGAEA.983965 (sedimentological and pore water data; Seemann, Jenrich, Lindemann, et al. 2025a), https://doi.pangaea.de/10.1594/PANGAEA.984832 (incubation data; Seemann, Baysinger, Liebner, et al. 2025), https://doi.pangaea.de/10.1594/PANGAEA.983966 (n‐Alkane biomarker data; Seemann, Jenrich, Lindemann, et al. 2025b), https://doi.pangaea.de/10.1594/PANGAEA.985099 (microbial data; Seemann, Liebner, Saborowski, Jenrich, et al. 2025).
