# Supplementary material for: Saline Permafrost and Cryopegs as Potentially Important Sources of CO2 —Assessing Organic Carbon Mineralization Potentials on the Alaskan Coastal Plain
Source: Glob Chang Biol. 2026 Jul 11;32(7):e70997. doi: 10.1111/gcb.70997 (PMC13355874; doi:10.1111/gcb.70997)
Supplement: Supplementary file 1 — Figure S1: CO2 production per gram dry weight (DW) and per gram total organic carbon (TOC) during the incubation experiment, including standard deviation (shaded areas). (a) UL samples, (b) West Twin Lake (WTL) and East Twin Lake (ETL) samples, (c) DLB samples (note the y‐axis break of the left panel). Table S1: CO2 production rates during 382 days of aerobic incubation, and days at which extreme values occurred. Table S2: Total n‐alkane content (TAC in μg g−1 TOC) before and after the aerobic incubation experiment of 382 days. [file GCB-32-e70997-s001.pdf]

Supplementary material to

Saline permafrost and cryopegs as potentially important sources of CO_2 - Assessing organic carbon mineralization potentials on the Alaskan Coastal Plain

Fabian Seemann[1,2,3], Mackenzie R. Baysinger[1], Susanne Liebner[4,5], Claire Treat[6], Michael Zech[3], Maren Jenrich[1], Guido Grosse[1,2], Benjamin M. Jones[7], and Jens Strauss[1]

[1] Permafrost Research Section, Alfred Wegener Institute Helmholtz Centre for Polar and Marine Research, Potsdam, Germany
[2] Institute of Geosciences, University of Potsdam, Potsdam, Germany
[3] Physical Geography, Institute of Geography, Technical University Dresden, Dresden, Germany
[4] GFZ Helmholtz Centre for Geosciences, Section Geomicrobiology, Potsdam, Germany
[5] Institute of Biochemistry and Biology, University of Potsdam, Potsdam, Germany
[6] Land-CRAFT Center, Department of Agroecology, Aarhus University, Aarhus, Denmark
[7] Institute of Northern Engineering, University of Alaska Fairbanks, Fairbanks, Alaska, USA

Correspondence: Fabian Seemann (fabian.seemann@awi.de; + 49 331 58174 5436)

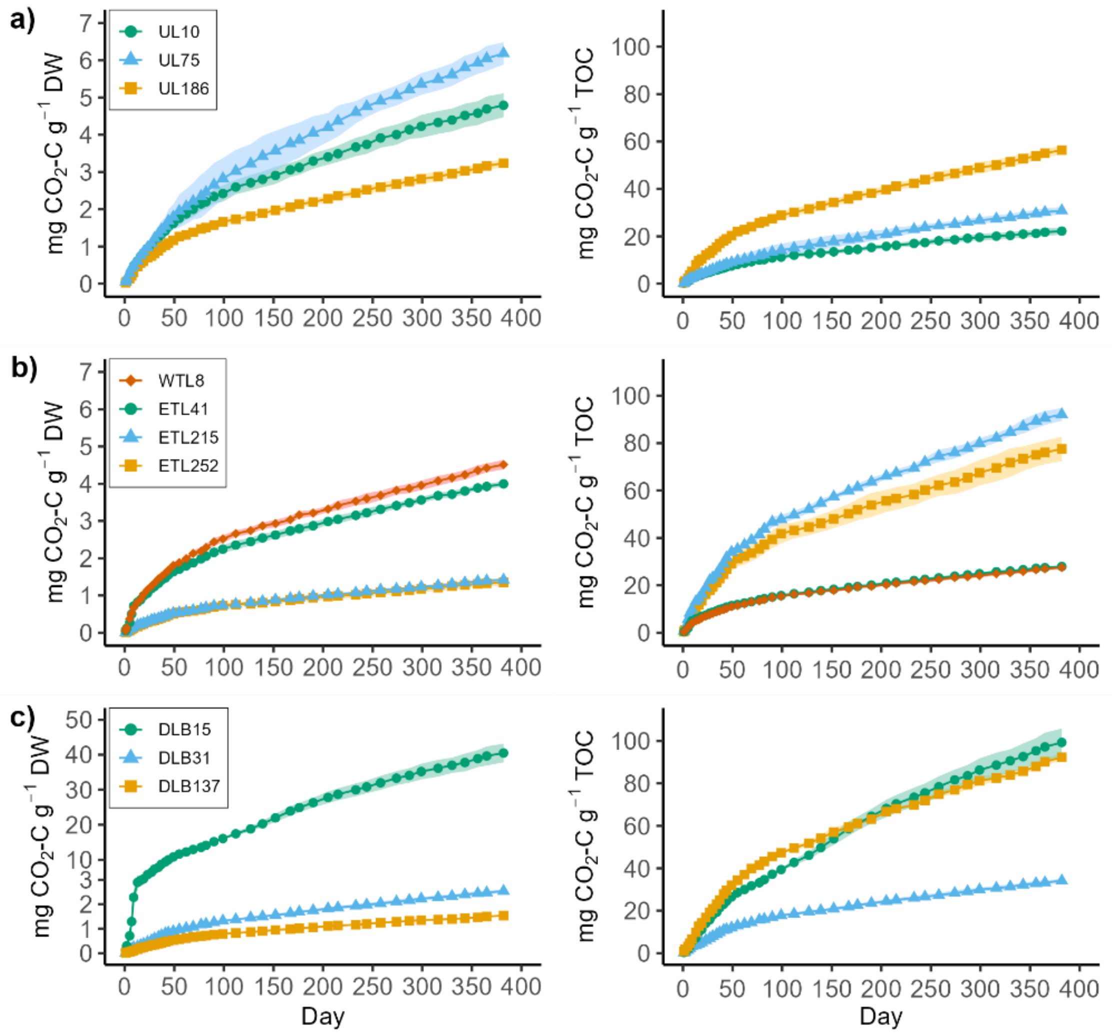

Figure S1: CO_2 production per gram dry weight (DW) and per gram total organic carbon (TOC) during the incubation experiment, including standard deviation (shaded areas). (a) UL samples, (b) West Twin Lake (WTL) and East Twin Lake (ETL) samples, (c) DLB samples (note the y-axis break of the left panel).

Table S1: CO_2 production rates during 382 days of aerobic incubation, and days at which extreme values occurred.

Sample	CO_2 production rate (mg C g^{-1} DW d^{-1})			CO_2 production rate (µg C g^{-1} TOC d^{-1})			Day of max/min production	
	max	min	mean	max	min	mean	max	min
UL10	0.086	0.005	0.020	0.400	0.022	0.092	9	330
UL75	0.084	0.007	0.024	0.419	0.037	0.121	2	316
UL186	0.045	0.004	0.014	0.787	0.063	0.239	9	316
WTL8	0.080	0.004	0.021	0.486	0.022	0.129	5	190
ETL41	0.115	0.003	0.020	0.805	0.019	0.138	7	330
ETL215	0.021	0.001	0.006	1.359	0.089	0.394	7	382
ETL252	0.020	0.001	0.006	1.131	0.084	0.336	7	274
DLB15	0.495	0.050	0.143	1.213	0.123	0.351	9	382
DLB31	0.035	0.003	0.011	0.468	0.042	0.143	7	365
DLB137	0.019	0.001	0.006	1.133	0.075	0.382	2	316

Table S2: Total *n*-alkane content (TAC in µg g^{-1} TOC) before and after the aerobic incubation experiment of 382 days.

Sample	pre (µg g^{-1} TOC)	post (µg g^{-1} TOC)	change (%)
UL10	524.7	1164.8	122
UL75	179.1	607.5	239
UL186	89.3	199.9	124
WTL8	1227.1	3907.9	218
ETL41	2065.8	6855.8	232
ETL215	129.7	228.0	76
ETL252	1020.0	592.0	-42
DLB15	31.4	188.2	499
DLB31	1448.9	1682.8	16
DLB137	91.9	86.6	-6
**Average**	**680.8**	**1551.4**	**148**
